# Study of *Helicobacter pylori* infection in patients with chronic atrophic gastritis and its relationship with lifestyle habits and dietary nutrient intake: A retrospective analysis

**DOI:** 10.1097/MD.0000000000036518

**Published:** 2024-01-12

**Authors:** Peilin Li, Weiqin Zhu, Jianhua Ding, Fenfang Lei

**Affiliations:** a Department of Nursing, Shaoyang University, Shaoyang, Hunan, China; b Department of General Surgery, The Second Affiliated Hospital of Shaoyang University, Shaoyang, Hunan, China.

**Keywords:** chronic atrophic gastritis, clinical characteristics, dietary factors, *Helicobacter pylori*, infection investigation, lifestyle habits

## Abstract

To explore *Helicobacter pylori* (Hp) infection status and its relationship with lifestyle habits and dietary factors in patients with chronic atrophic gastritis. Six hundred thirty-eight patients with chronic atrophic gastritis, who were admitted to our hospital from March 2021 to April 2023, were selected for the study. All patients underwent the ^13^C urea breath test. The relationship between the detection rate of Hp infection and the clinical characteristics, lifestyle habits, and dietary factors of the patients was analyzed. Among the 638 patients with chronic atrophic gastritis, 531 patients were tested positive for Hp infection, the positive rate for Hp infection was approximately 83.23%. Analyzing the clinical characteristics of the patients, it was found that age, family history of gastric cancer, degree of chronic inflammation, degree of glandular atrophy, presence of low-grade dysplasia, and intestinal metaplasia all have an impact on the positive detection rate of patients (*P* < .05). Analyzing the patients’ lifestyle habits, it was found that BMI, smoking history, alcohol consumption, preference for spicy food, dining location, consumption of pickled foods, frequent consumption of grilled/barbecued foods, preference for strong tea, consumption of sweets, and work-related stress had an impact on the positive rate of Hp infection in patients (*P* < .05). The discovery showed that the levels of total protein, albumin, hemoglobin, cholesterol, and the intake of livestock and poultry meat, seafood, dairy products, vegetables, fruits, and fats have an impact on the positivity rate of Hp infection in patients (*P* < .05). A multiple logistic regression analysis was performed, and it was found that patients’ age, family history of gastric cancer, degree of chronic inflammation, degree of glandular atrophy, presence of low-grade dysplasia, presence of wasting or obesity, history of alcohol consumption, preference for spicy food, dining location, frequent consumption of strong tea, high work pressure, high intake of fish and seafood, low intake of dairy products, low intake of vegetables, low intake of fruits, and low intake of fats all had an impact on the occurrence of Hp infection in patients (*P* < .05). There is a certain correlation between patients’ lifestyle habits, dietary factors, and clinical characteristics with the occurrence of Hp infection. These factors can assist in the prevention of Hp infection.

## 1. Introduction

Chronic atrophic gastritis represents a prevalent precancerous condition affecting the stomach. It is characterized by persistent inflammation and tissue atrophy of the gastric mucosa.^[1]^ This condition is associated with various factors, including infection with *Helicobacter pylori* (Hp), dietary habits, and familial inheritance.^[2]^ Hp is a microaerophilic Gram-negative bacterium that was first isolated from live gastric mucosal biopsy specimens of patients with chronic active gastritis in 1983. Hp has a distinctive spiral shape and flagella, which enable it to attach to the surface of the gastric mucosa and penetrate the mucus layer, colonizing the surface epithelial cells of the gastric mucosa. Hp exhibits strong survival capabilities and can adapt to the gastric acid environment and host defense mechanisms, leading to chronic inflammation of the gastric mucosa and potentially causing various gastric diseases, including chronic gastritis, gastric ulcers, and gastric cancer.^[3,4]^ Beyond its close association with gastric diseases, Hp also influences the body’s nutritional status. Past research has primarily concentrated on assessing the link between individual foods or their constituents and the risk of Hp infection. However, comprehensive investigations into lifestyle practices and overall dietary patterns have been relatively scarce.^[1]^ Consequently, this present study aims to broaden the spectrum of influencing factors, delving into the Hp infection status among chronic atrophic gastritis patients, along with its correlation with lifestyle habits and dietary elements.

## 2. Data and methods

### 2.1. General data

This study was approved by the Ethics Committee of Shaoyang University. Six hundred thirty-eight patients with chronic atrophic gastritis, admitted to our hospital from March 2021 to April 2023, were included in this study. All patients underwent the ^13^C urea breath test, and the results of the test were confirmed. Inclusion criteria: ① patients were over 18 years old. ② According to the “Chinese Consensus on Chronic Gastritis,”^[5]^ the patient was diagnosed with chronic atrophic gastritis. ③ Patients with complete clinical data, living habits, nutrition and eating habits can be studied.

Exclusion criteria: ① the patient had other serious gastrointestinal digestive system diseases. ② Female patients in lactation or pregnancy. ③ Patients had recently taken antibiotics or proton pump inhibitors and other drugs. ④ There were serious liver and kidney lesions. ⑤ Irritable bowel syndrome.

### 2.2. Methods

*Hp testing*: Before the test, patients are required to fast for at least 8 hours or have an empty stomach. Within 24 hours prior to the test, patients should avoid using antibiotics, gastric medications, and bismuth-containing drugs. Subjects should first remove the cap from the end of the breath bag, then open the blowing tube and ensure a secure connection to the bag for an airtight seal. They will be instructed to exhale slowly into the breath bag while maintaining normal breathing. Once the bag is filled, subjects should grip it tightly with both hands, remove the blowing tube, and swiftly seal the airbag port using the small cap to prevent gas leakage. This step collects baseline gas before medication. Next, subjects will receive approximately 20 milliliters of warm water and ingest 1 C-urea tablet. They must remain seated for about 20 to 25 minutes. After medication, follow the same procedure to collect exhaled breath samples. The collected breath samples are then placed in an HG-IRI-C-13C infrared spectrometer for CO_2_ detection. The instrument analyzes and calculates the difference in carbon dioxide enrichment of ^13^C atoms between the post-medication and baseline exhaled gases, known as the Delta Over Baseline.

*Questionnaire survey*: A self-designed questionnaire developed by the department was utilized for the survey. The questionnaire underwent review by professional staff within the hospital and was distributed to the participants following standardized procedures. Before the survey commenced, surveyors engaged in one-on-one communication with each respondent. They provided a detailed explanation of the main content and purpose of the survey, including the background, research objectives, and confidentiality. After obtaining the patients’ consent, the questionnaire was administered for completion. The survey included the following demographic and lifestyle factors: age, gender, presence of diabetes, family history of gastric cancer, educational level, BMI, smoking history, alcohol consumption, preference for spicy food, preference for dietary habits, dining location, frequency of consuming pickled foods, frequency of consuming grilled/barbecued foods, source of drinking water, regular consumption of strong tea, preference for sweet foods, work stress level, daily intake (in grams) of grains and potatoes, intake of cereals and tubers, meat and poultry, fish and shellfish, legumes, dairy products, eggs, vegetables, fruits, and oils. The degree of chronic inflammation, extent of glandular atrophy, and presence of gastric mucosal intestinal metaplasia were collected based on the patients’ gastroscopy examination results.

The measurements of total protein, albumin, hemoglobin, and cholesterol were conducted by collecting venous blood samples from the patients after a fasting period of 6 to 8 hours. The blood samples were centrifuged at a radius of 10 cm, a speed of 3500 r/min, and a duration of 13 minutes. The obtained serum specimens were then analyzed using the AU5800 fully automated biochemical analyzer manufactured by Beckman Coulter.

### 2.3. Observation indicators

All patients underwent the ^13^C urea breath test to analyze the correlation between different clinical characteristics, lifestyle habits, dietary nutrition factors, and their Hp test results.

*Hp positive criteria*: The difference in the abundance of ^13^C-labeled carbon dioxide before and after medication is recorded, and a difference value of not <4 is considered positive for Hp, otherwise it is considered negative.

Statistical analysis was conducted to measure the levels of serum total protein, albumin, hemoglobin, and cholesterol in the patients.

### 2.4. Statistical processing

The data processing for this study was conducted using SPSS 23.0 software. Various count data related to the patients, such as their past medical history, age, years of education, family history of gastric cancer, degree of chronic inflammation, degree of glandular atrophy, presence of low-grade dysplasia, and intestinal metaplasia, were subjected to a Chi-square test. The results were expressed as counts (n) and percentages (%). For measurement data, including the intake of dietary nutrition factors and serum levels of total protein, albumin, hemoglobin, cholesterol, and other groups, independent sample *t*-tests were utilized for analysis. Intragroup comparisons were performed using paired *t*-tests. The results for measurement data were expressed as mean (± standard deviation). The significance level for all tests was set at α = 0.05.

## 3. Results

### 3.1. *Helicobacter pylori* infection in patients with chronic atrophic gastritis

Among the 638 patients with chronic atrophic gastritis included in the study, all of them underwent the 13C urea breath test. Among these 531 patients, all of them tested positive for Hp infection, resulting in a positive detection rate of approximately 83.23%.

### 3.2. The relationship between the clinical characteristics of patients and the results of Hp examination

The analysis of clinical characteristics showed that gender, diabetes, and education level were not statistically associated with the Hp detection rate among the patients (*P* > .05). However, age, family history of gastric cancer, degree of chronic inflammation, degree of glandular atrophy, presence of low-grade dysplasia, and intestinal metaplasia were found to significantly influence the positive detection rate of Hp among the patients (*P* < .05). For more details, please refer to Table 1.

**Table 1:** The relationship between the clinical characteristics of patients and the results of Hp examination (n/%).

Clinical characteristics	Number of cases examined	Hp check result		Detection rate (%)	χ^2^	*P*
		Positive	Negative			
*Age (years*)						
<60	267	212	55	79.40	4.820	**.028**
≥60	371	319	52	85.98		
*Sex*						
Male	324	265	59	81.79	0.976	.323
Female	314	266	48	84.71		
*Diabetes mellitus*						
Yes	231	198	33	85.71	1.603	.206
No	407	333	74	81.82		
*Family history of gastric cancer*						
Yes	462	398	64	86.15	10.218	**.001**
No	176	133	43	75.57		
*Years of schooling (years*)						
≥12	276	222	54	80.43	2.721	.099
<12	362	309	53	85.36		
*Chronic inflammation*						
Mild and moderate	243	156	87	64.20	101.843	**<.001**
Severe	395	375	20	94.94		
*Glandular atrophy*						
Mild and moderate	280	205	75	73.21	35.853	**<.001**
Severe	358	326	32	91.06		
*Low-grade dysplasia*						
Yes	216	200	16	92.59	20.513	**<.001**
No	422	331	91	78.44		
*Intestinal metaplasia*						
Yes	220	198	22	90.00	11.030	**.001**
No	418	333	85	79.67		

Bold values indicate statistically significant.

### 3.3. The relationship between patients’ living habits and Hp test results

The analysis of patients’ lifestyle habits indicated that BMI, smoking history, alcohol consumption, preference for spicy food, dining location, consumption of pickled food, frequent consumption of barbecued food, consumption of strong tea, intake of sweets, and high work stress all significantly influenced the positive detection rate of Hp among the patients (*P* < .05) (Table 2).

**Table 2:** The relationship between patients’ living habits and Hp test results (n/%).

Living habit	Number of cases examined	Hp check result		Detection rate (%)	χ^2^	*P*
		po	negative			
BMI						
Normal	293	212	81	72.35	45.899	**<.001**
Wasting or obesity	345	319	26	92.46		
*Smoking history*						
Yes	394	339	55	86.04	5.835	**.016**
No	244	192	52	78.69		
*Drinking history*						
Yes	334	288	46	86.23	4.516	**.034**
No	304	243	61	79.93		
*Love spicy food*						
Yes	374	337	37	90.11	30.633	**<.001**
No	264	194	70	73.48		
*Vegetarian*						
Yes	300	256	44	85.33	1.797	.180
No	338	275	63	81.36		
*Dining place*						
Living at home	204	115	89	56.37	154.959	**<.001**
Canteen or restaurant	434	416	18	95.85		
*Salted food*						
Frequently	326	311	15	95.40	70.733	**<.001**
Rarely or never	312	220	92	70.51		
*Barbecue*						
Frequently	220	198	22	90.00	11.030	**.001**
Rarely or never	418	333	85	79.67		
*Source of drinking water*						
Tap water	380	335	45	88.16	16.356	**<.001**
Underground water	258	196	62	75.97		
*Drink strong tea regularly*						
Yes	314	289	25	92.04	34.376	**<.001**
No	324	242	82	74.69		
*Have a sweet tooth*						
Yes	328	294	34	89.63	19.842	**<.001**
No	310	237	73	76.45		
*Work strain*						
Yes	353	326	27	92.35	47.112	**<.001**
No	285	205	80	71.93		

Bold values indicate statistically significant.

### 3.4. The relationship between dietary nutritional factors and Hp test results in patients

The analysis of dietary nutrition factors and their correlation with serum total protein, albumin, hemoglobin, and cholesterol between Hp-positive and Hp-negative patients demonstrated that serum total protein, albumin, hemoglobin, cholesterol, as well as the consumption of poultry and meat, fish and seafood, dairy products, vegetables, fruits, and fats, all had a significant influence on the positive detection rate of Hp among the patients (*P* < .05). Please refer to Table 3 for more detailed information.

**Table 3:** The relationship between dietary nutritional factors and Hp test results in patients (±s).

Dietary nutrition factor	Hp check result		*t*	*P*
	Positive (n = 531)	Negative (n = 107)		
TP (total protein) (g/L)	57.66 ± 6.56	63.45 ± 8.78	−7.829	**<.001**
Albumin (g/L)	31.26 ± 3.45	35.66 ± 3.16	−12.200	**<.001**
Hemoglobin (g/L)	85.16 ± 11.02	93.45 ± 13.04	−6.874	**<.001**
Cholestenon (mmol/L)	4.02 ± 1.78	3.45 ± 0.98	3.214	**.001**
Yams (g/d)	422.21 ± 121.21	411.26 ± 118.29	0.856	.392
Poultry and meat (g/d)	66.78 ± 21.11	72.13 ± 33.15	−2.144	**.032**
Fatherlasher (g/d)	21.48 ± 5.26	18.99 ± 5.44	4.442	**<.001**
Beans (g/d)	20.68 ± 6.59	21.45 ± 4.27	−1.160	.246
Flesvoeding (g/d)	60.56 ± 14.32	65.44 ± 8.97	−3.392	**.001**
Eggs (g/d)	55.26 ± 7.26	55.28 ± 7.22	−0.026	.979
Vegetables (g/d)	298.24 ± 45.09	333.16 ± 38.12	−7.829	**<.001**
Fruit (g/d)	92.17 ± 8.59	118.24 ± 7.64	−12.200	**<.001**
Grease (g/d)	28.99 ± 3.23	36.84 ± 6.55	−6.874	**<.001**

Bold values indicate statistically significant.

### 3.5. Influencing factors of Hp test results in patients

Logistic regression analysis was conducted to investigate the relationship between the positive detection rate of Hp (positive = 1, negative = 0) and various factors, including patient age (<60 years = 0, ≥60 years = 1), family history of gastric cancer (present = 0, absent = 1), severity of chronic inflammation (mild to moderate = 0, severe = 1), degree of glandular atrophy (mild to moderate = 0, severe = 1), presence of low-grade dysplasia (present = 0, absent = 1), intestinal metaplasia (present = 0, absent = 1), BMI (normal = 0, obese or underweight = 1), smoking history (yes = 0, no = 1), alcohol consumption history (yes = 0, no = 1), preference for spicy food (yes = 0, no = 1), dining location (home = 0, canteen or restaurant = 1), consumption of pickled food (rarely or never = 0, often = 1), consumption of grilled food (rarely or never = 0, often = 1), source of drinking water (tap water = 0, groundwater = 1), regular consumption of strong tea (yes = 0, no = 1), preference for sweet food (yes = 0, no = 1), work stress (yes = 0, no = 1), as well as the levels of total protein, albumin, hemoglobin, cholesterol, consumption of fish and seafood, dairy products, vegetables, fruits, and fats. The analysis revealed that patient age, family history of gastric cancer, severity of chronic inflammation, degree of glandular atrophy, presence of low-grade dysplasia, obesity or underweight, alcohol consumption history, preference for spicy food, dining location, regular consumption of strong tea, work stress, consumption of fish and seafood, low consumption of dairy products, low consumption of vegetables, low consumption of fruits, and low consumption of fats all significantly influenced the risk of Hp infection among the patients (*P* < .05) (Table 4).

**Table 4:** Influencing factors of Hp test results in patients.

Element	Unnormalized coefficient		Beta	95.0% confidence interval		*t*	*P*
	B	Standard error		Floor	Upper limit		
(constant)	0.831	0.031	–	0.771	0.891	27.162	**<.001**
Age	−0.067	0.033	−0.088	−0.131	−0.003	−2.045	**.041**
Family history of gastric cancer	0.089	0.033	0.107	0.024	0.155	2.686	**.007**
Degree of chronic inflammation	0.366	0.036	0.475	0.295	0.436	10.187	**<.001**
The degree of gland atrophy	0.508	0.047	0.675	0.415	0.601	10.738	**<.001**
Low-grade dysplasia	−0.771	0.095	−0.977	−0.958	−0.585	−8.113	**<.001**
Intestinal	−0.039	0.095	−0.050	−0.226	0.147	−0.415	.678
BMI	−0.078	0.028	−0.104	−0.133	−0.024	−2.830	**.005**
History of smoking	0.050	0.028	0.059	−0.005	0.104	1.774	.077
History of drinking	0.119	0.035	0.155	0.050	0.188	3.373	**.001**
Love to eat spicy	0.180	0.048	0.240	0.086	0.274	3.771	**.000**
Dining place	−0.318	0.096	−0.403	−0.506	−0.130	−3.327	**.001**
Pickled food	−0.017	0.094	−0.022	−0.201	0.167	−0.183	.855
Barbecue	−0.084	0.062	−0.084	−0.206	0.037	−1.361	.174
Source of drinking water	0.018	0.059	0.018	−0.097	0.134	0.313	.755
Drink plenty of tea regularly	0.178	0.049	0.238	0.081	0.278	3.778	**.003**
Have a sweet tooth	−0.006	0.100	−0.006	−0.202	0.190	−0.062	.950
Work strain	0.702	0.057	0.702	0.589	0.814	12.220	**.000**
Total protein	−0.114	0.071	−0.114	−0.254	0.026	−1.593	.112
Albumin	0.031	0.110	0.031	−0.184	0.246	0.286	.775
Hemoglobin	−0.019	0.156	−0.019	−0.326	0.289	−0.119	.905
Cholesterin	−0.015	0.145	−0.018	−0.317	0.238	−0.115	.815
Fish and shrimp	−0.056	0.015	−0.313	−0.087	−0.019	−3.449	**.008**
Milk class	0.118	0.046	0.166	0.053	0.198	3.369	**.015**
Green grocery	1.185	0.411	1.161	0.367	2.003	2.880	**.005**
Fruit class	−0.006	0.003	−0.229	−0.012	−0.001	−2.370	**.020**
Lipa	−0.059	0.017	−0.323	−0.094	−0.025	−3.420	**.001**

Bold values indicate statistically significant.

## 4. Discussion

Hp belongs to a group of microaerophilic Gram-negative rod bacteria. It primarily resides in the gastric mucosal layer. Hp infection typically spreads through oral–oral or oral–fecal routes and is commonly transmitted through sources like food, water, household contacts, or close interpersonal interactions.^[6,7]^ Numerous studies^[8–11]^ have demonstrated a significant correlation between Hp and various gastrointestinal disorders, including chronic atrophic gastritis. The primary pathological mechanism involves chronic inflammation of the gastric mucosa triggered by Hp infection, leading to gastric mucosal atrophy and functional impairments. Consequently, this leads to decreased gastric acid secretion, gastric mucosal atrophy, reduced digestive enzyme activity, and the emergence of symptoms such as upper abdominal pain, bloating, loss of appetite, as well as weight loss and anemia.^[12,13]^ Studies have shown that, in cases of Hp infection, if left untreated, approximately 5% of infected individuals may progress to gastric mucosal atrophy, resulting in chronic atrophic gastritis. However, successful eradication of Hp infection significantly improves the cure rate of Hp-related gastric inflammation.^[14]^ Besides Hp infection, the development of chronic atrophic gastritis is influenced by various factors, including patients’ dietary habits, nutritional intake, and genetic factors.^[15]^ Therefore, investigating the prevalence of Hp infection in patients with chronic atrophic gastritis and its correlation with lifestyle habits and dietary factors holds significant importance for the prevention and management of chronic atrophic gastritis and associated conditions.

This study analyzed the prevalence of Hp infection in patients with chronic atrophic gastritis, and the results showed that the positive detection rate of Hp infection in the study population was approximately 83.23% (531/638). This result suggests that in patients with chronic atrophic gastritis, there is a higher prevalence of Hp infection compared to the local Hp infection rate of 40 to 60%. This study further analyzed the relationship between clinical characteristics of patients and their Hp examination results. The results showed that age, family history of gastric cancer, severity of chronic inflammation, degree of glandular atrophy, presence of low-grade dysplasia, and intestinal metaplasia all had a significant impact on the positive detection rate of Hp infection in patients (*P* < .05). The specific reasons are as follows: aging is usually associated with a decline in immune system function, reduced gastric acid secretion, and long-term exposure to risk factors for Hp infection. A family history of gastric cancer may be related to genetic factors and a high degree of similarity in shared environment. Individuals with a family history of gastric cancer are more likely to carry Hp infection.^[16]^ Chronic atrophic gastritis is an important pathological basis for Hp infection. The more severe the inflammation, the more severe the damage to the gastric mucosa, and therefore the higher the likelihood of Hp infection. In addition, glandular atrophy is a typical feature of chronic atrophic gastritis. The loss of glands and reduced gastric acid secretion create an environment where Hp can survive and proliferate, increasing the risk of Hp infection.^[17]^ Intestinal metaplasia is a manifestation of abnormal proliferation and variation of the gastric mucosa. It is considered a precancerous lesion of gastric cancer. The presence of intestinal metaplasia indicates abnormal changes in the gastric mucosa, providing a more suitable environment for Hp infection. The logistic regression analysis in Table 4 of this study further confirms the correlation between these factors and Hp infection.^[18]^ Intestinal metaplasia refers to the phenomenon where the gastric mucosal epithelial cells transform into a structure resembling intestinal epithelium. This may lead to a decrease in gastric mucosal acidity. However, the intestinal metaplasia of the gastric mucosa is influenced by multiple factors, and the specific mechanism of its impact on Hp infection requires further research.^[19]^

Studies have indicated that Hp infection is one of the main causes of chronic atrophic gastritis. Lifestyle habits can influence immune function, gastric acid secretion, and the protective barrier of the gastric mucosa, which may have an impact on the occurrence of Hp infection and its detection results. The results of this study indicated that several lifestyle habits of the patients, including BMI, smoking history, alcohol consumption, preference for spicy food, dining location, consumption of pickled foods, regular consumption of barbecued food, preference for strong tea, consumption of sweet food, and work stress, all had a significant impact on the Hp positivity rate in patients (*P* < .05). This suggested that these lifestyle factors may be associated with an increased Hp positivity rate in patients. However, subsequent logistic regression analysis revealed that factors such as being underweight or overweight, alcohol consumption, preference for spicy food, dining location, regular consumption of strong tea, and work stress were more closely associated with Hp infection. This was because being underweight or overweight is related to the overall nutritional status and immune function of patients. Factors such as compromised immune system and metabolic disorders can increase the risk of Hp infection. Similarly, long-term work stress can also affect the immune function of patients, leading to weakened resistance and making the body more susceptible to Hp infection.^[20,21]^ Additionally, alcohol consumption and the consumption of spicy foods, known for their high stimulation, can increase stomach acid secretion and irritate the gastric mucosa. This compromises the protective barrier of the mucosa and makes it more susceptible to Hp invasion.^[22]^ The choice of dining location may also be related to food hygiene. Dining at external establishments with inadequate utensil disinfection and crowded eating environments align closely with the transmission routes of Hp in the digestive system, thus increasing the risk of Hp infection. Substances like caffeine and tannic acid in strong tea can stimulate the gastric mucosa, leading to increased stomach acid secretion and, consequently, an elevated risk of Hp infection.^[23,24]^

The study also delved into the relationship between dietary nutrition factors and Hp test results. The findings highlighted that serum total protein, albumin, hemoglobin, cholesterol, consumption of poultry and livestock meat, fish and shrimp, dairy products, vegetables, fruits, and fats all had a significant impact on the Hp positivity rate in patients (*P* < .05). This underscores the close connection between protein intake, dietary fiber, and vital nutrients like vitamins with the likelihood of Hp infection. Further logistic regression analysis indicated that higher consumption of fish and shellfish, lower consumption of dairy products, lower consumption of vegetables, lower consumption of fruits, and lower consumption of fats were all associated with an increased risk of Hp infection in patients (*P* < .05). This is primarily because vegetables and fruits are essential sources of dietary fiber, which plays a critical role in regulating gastrointestinal function and maintaining intestinal health. A sufficient intake of dietary fiber can promote regular bowel movements, reduce the risk of gastric acid reflux, mucosal damage, and aid in preventing Hp infection.^[25,26]^ Vegetables and fruits are also rich sources of various vitamins, such as vitamin C and vitamin E. Vitamins have antioxidant and anti-inflammatory effects, which help protect the gastric mucosa from damage. Inadequate intake of vegetables and fruits may result in vitamin deficiencies and weaken the mucosal barrier function, thereby increasing the risk of Hp infection.^[27–29]^ Fish and shellfish are known for their high content of omega-3 fatty acids, while dairy products provide ample amounts of calcium and vitamin D. Omega-3 fatty acids exhibit anti-inflammatory and antimicrobial effects, which assist in reducing gastric mucosal inflammation and maintaining gastrointestinal health.^[12,30]^ Additionally, moderate fat intake is crucial for safeguarding the gastric mucosa and preserving the integrity of the mucosal barrier. Fats play a key role as carriers of fat-soluble vitamins, aiding in the absorption of these vitamins and maintaining the normal functioning of the mucosal barrier.^[31–33]^ The above conclusions suggested that in the prevention and management of chronic atrophic gastritis, attention should be paid to patients’ lifestyle habits and dietary nutrient intake. Appropriate interventions should be taken to reduce the risk of Hp infection.

In summary, patients’ lifestyle habits, dietary nutrient intake, and clinical characteristics are associated with Hp infection and can be used as supportive measures in the prevention of Hp infection.

## 5. Limitation

This study has certain limitations and future prospects. Firstly, due to geographical constraints, this research did not conduct a multicenter study, which may introduce regional limitations, such as the prevalence of dietary habits and preferences in specific geographic areas. However, we made efforts to ensure data balance during collection and analysis to maintain the representativeness of the data. We are also planning to conduct a multicenter study in the future to broaden the scope of the research. Secondly, in terms of data collection, as this study is retrospective, there were some issues related to missing data, particularly in the dietary factors. Not all dietary preferences were included, and we retained factors with more complete data. We intend to comprehensively enhance data collection, especially regarding medical histories, in subsequent cases to conduct more thorough analyses. Finally, we plan to conduct specific subtyping research based on clinical indicators, behaviors, and dietary habits that have shown statistical significance in this study. This will help us determine whether these indicators exhibit tendencies in different disease subtypes. Our goal is to refine the results of this study to better cater to the needs of various population groups.

## Author contributions

**Conceptualization:** Peilin Li, Weiqin Zhu, Fenfang Lei.

**Data curation:** Peilin Li, Fenfang Lei.

**Formal analysis:** Peilin Li, Weiqin Zhu, Jianhua Ding, Fenfang Lei.

**Investigation:** Weiqin Zhu, Jianhua Ding.

**Methodology:** Jianhua Ding, Fenfang Lei.

**Software:** Jianhua Ding.

**Supervision:** Weiqin Zhu, Fenfang Lei.

**Writing – original draft:** Peilin Li.

**Writing – review & editing:** Peilin Li, Fenfang Lei.
